# Evaluation of Microsatellite Typing, ITS Sequencing, AFLP Fingerprinting, MALDI-TOF MS, and Fourier-Transform Infrared Spectroscopy Analysis of *Candida auris*

**DOI:** 10.3390/jof6030146

**Published:** 2020-08-25

**Authors:** Mansoureh Vatanshenassan, Teun Boekhout, Norman Mauder, Vincent Robert, Thomas Maier, Jacques F. Meis, Judith Berman, Euníce Then, Markus Kostrzewa, Ferry Hagen

**Affiliations:** 1 Bruker Daltonik GmbH, 28359 Bremen, Germany; m.vatanshenassan@wi.knaw.nl (M.V.); norman.mauder@bruker.com (N.M.); thomas.maier@bruker.com (T.M.); 2 Westerdijk Fungal Biodiversity Institute, 3584 CT Utrecht, The Netherlands; t.boekhout@wi.knaw.nl (T.B.); v.robert@wi.knaw.nl (V.R.); e.then@wi.knaw.nl (E.T.); 3 Institute for Biodiversity and Ecosystem Dynamics (IBED), University of Amsterdam, 1012 WX Amsterdam, The Netherlands; 4 BioAware, B-4280 Hannut, Belgium; 5 Department of Medical Microbiology and Infectious Diseases, Canisius Wilhelmina Hospital (CWZ), 6532 SZ Nijmegen, The Netherlands; jacques.meis@gmail.com; 6 Center of Expertise in Mycology Radboudumc, Canisius Wilhelmina Hospital (CWZ), 6532 SZ Nijmegen, The Netherlands; 7 Bioprocess Engineering and Biotechnology Graduate Program, Federal University of Paraná, 80060-000 Curitiba, Brazil; 8 Shmunis School of Biomedicine and Cancer Research, George S. Wise Faculty of Life Sciences, 6997801 Tel Aviv, Israel; judithberman11@gmail.com; 9 Department of Medical Microbiology, University Medical Center Utrecht, 3584 CX Utrecht, The Netherlands

**Keywords:** *Candida auris*, molecular epidemiology, epidemiological typing, nosocomial outbreak

## Abstract

*Candida auris* is an emerging opportunistic yeast species causing nosocomial outbreaks at a global scale. A few studies have focused on the *C. auris* genotypic structure. Here, we compared five epidemiological typing tools using a set of 96 *C. auris* isolates from 14 geographical areas. Isolates were analyzed by microsatellite typing, ITS sequencing, amplified fragment length polymorphism (AFLP) fingerprint analysis, matrix-assisted laser desorption/ionization-time of flight mass spectrometry (MALDI-TOF MS), and Fourier-transform infrared (FTIR) spectroscopy methods. Microsatellite typing grouped the isolates into four main clusters, corresponding to the four known clades in concordance with whole genome sequencing studies. The other investigated typing tools showed poor performance compared with microsatellite typing. A comparison between the five methods showed the highest agreement between microsatellite typing and ITS sequencing with 45% similarity, followed by microsatellite typing and the FTIR method with 33% similarity. The lowest agreement was observed between FTIR spectroscopy, MALDI-TOF MS, and ITS sequencing. This study indicates that microsatellite typing is the tool of choice for *C. auris* outbreak investigations. Additionally, FTIR spectroscopy requires further optimization and evaluation before it can be used as an epidemiological typing method, comparable with microsatellite typing, as a rapid method for tracing nosocomial fungal outbreaks.

## 1. Introduction

*Candida auris* was described in 2009 in Japan and since then it has caused infections at a global scale with a serious nosocomial health risk [1,2,3]. In less than a decade, *C. auris* was isolated on all six human-inhabited continents [4,5], and infections were reported from more than 40 countries [4,6,7]. Nosocomial *C. auris* outbreaks were first reported from South Korea [8], followed by India [9,10], South Africa [11], Kuwait [12], Venezuela [13], USA [14], and European countries [2,3,7]. The phylogeographic structure of *C. auris* has been studied using whole genome sequencing (WGS) and internal transcribed spacer (ITS) sequencing. These studies suggested that the *C. auris* isolates belong to four separate geographic clades, namely Eastern Asia (Korea and Japan), Southern Asia (India and Pakistan), South Africa, and South America (Venezuela). Importantly, a fifth lineage was detected recently from a patient in Iran that never traveled outside the country. This Iranian isolate possessed >200,000 single-nucleotide polymorphisms (SNPs) relative to isolates from the other four clades and, thus, may represent a fifth clade [15,16]. Isolates belonging to the four currently recognized clades differ from each other by tens to hundreds of thousands of SNPs, and exhibit limited within-clade diversity of only tens of base pairs [1,17]. However, the isolates within one clade with limited genomic diversity may have broad physiological variety [17,18]. Therefore, using different genotyping and biochemical techniques could present a clear overview of the geographical distribution of typing *C. auris* isolates. A few studies have investigated the geographical distribution of *C. auris* by other genotyping techniques, such as amplified fragment length polymorphism (AFLP) fingerprinting [2,13,18] and microsatellite typing [19]. The latter was used for earlier population structure analysis of several pathogenic *Candida* species [19,20,21,22]. ITS sequencing was used not only for the identification of *Candida* isolates, but also for typing purposes [17,18,23,24]. Matrix-assisted laser desorption/ionization-time of flight mass spectrometry (MALDI-TOF MS) is widely used for the rapid and accurate identification of yeasts, including *C. auris* [18,25,26,27,28,29,30], and is also applied to *C. auris* strain typing [18,27,31]. The IR Biotyper (Bruker Daltonics, Bremen, Germany) was recently introduced into the field of microbial strain typing. This rapid and straightforward system is based on Fourier-transform infrared (FTIR) spectroscopy and uses molecular vibration fingerprints, primarily of the C-O stretching of biomacromolecules, e.g., carbohydrates, to characterize a microbial sample by strain-specific absorbance patterns in the infrared spectrum [32]. The IR Biotyper was recently developed for clinical applications, such as the study of nosocomial outbreaks and their dynamics, in order to prevent the spread of pathogens inside the hospitals.

FTIR spectroscopy had been applied to type isolates of *Saccharomyces cerevisiae* and was able to differentiate between different *Saccharomyces* species [33], but also for the analysis of cell wall structures [34,35]. The application of this method to type bacterial species in hospital outbreaks was investigated several times. For instance, typing clinical *Klebsiella* isolates [32,36] and Gram-negative bacilli clones was successfully performed using FTIR spectroscopy (IR Biotyper) [37]. A few studies have examined the role of FTIR spectroscopy in the typing of clinical isolates [38].

In this study, we compared different typing techniques, namely microsatellite typing, AFLP fingerprinting, ITS sequencing, MALDI-TOF MS, and IR Biotyper FTIR spectroscopy, to evaluate their application in typing *C. auris* isolates to further contribute to their epidemiology and dispersal routes in order to improve hospital hygiene and patient management.

## 2. Materials and Methods

### 2.1. *Candida auris* Isolates and Media

Isolates used in this study were collected from different geographical regions, and belonged to the previously defined four major clades based on molecular studies [1,14,17,19,26,39]. A set of 96 *C. auris* isolates was obtained from the Westerdijk Fungal Biodiversity Institute, Utrecht, The Netherlands (*n* = 37), the Centers for Disease Control and Prevention (CDC, Atlanta, GA, USA; *n* = 10), and individual researchers (*n* = 49). The majority of isolates came from India (*n* = 38) [2,9,10], followed by 58 isolates from other geographical areas, namely Austria (from a Turkish patient) (*n* = 1) [40], Belgium (from a Kuwaiti patient) (*n* = 2) [41], Japan (*n* = 3) [42], Israel (*n* = 9) [43,44], Korea (*n* = 3) [8], Malaysia (*n* = 3) [45], Oman (*n* = 13) [46,47], Pakistan (*n* = 2), Saudi Arabia (*n* = 2) [48], Spain (*n* = 13) [49], South Africa (*n* = 4), Switzerland (*n* = 1) [50], and Venezuela (*n* = 2) [13] (Supplementary Table S1). The isolates were identified by MALDI-TOF MS (Bruker Daltonik) using routine settings [27,51], and the Bruker MBT Compass Library, Revision E (7854 reference entries). The isolates were stored at −80 °C and cultured onto Sabouraud dextrose agar (SDA) overnight at 37 °C before further analysis.

### 2.2. Microsatellite Typing

All 96 strains were subjected to a recently developed 12-loci microsatellite typing scheme [19]. Genomic DNA of all *C. auris* isolates was extracted by the CTAB method as described elsewhere [52]. The twelve loci were amplified using monoplex PCR, and forward primers were all fluorescently labelled with fluorescein for subsequent detection by capillary electrophoresis on an ABI3730xL Genetic Analyzer platform (Applied Biosystems, Thermo Fisher Scientific, Foster City, CA, USA). Raw data were processed using BioNumerics v7.6 (Applied Maths, Sint-Martens-Latem, Belgium) and a UPGMA dendrogram was generated with BioloMICS v12 (BioAware, Hannut, Belgium).

### 2.3. Internal Transcribed Spacer Region (ITS) Sequencing

The amplification of the internal transcribed spacer (ITS) region was performed using primers ITS1 and ITS4, followed by Sanger sequencing using the same primers, as described elsewhere [44]. Raw sequence data were checked and contigs were compiled with SeqMan v12 (DNASTAR, Madison, WI, USA). BioloMICS v12 (BioAware) was used to analyze sequence data and create a UPGMA dendrogram. ITS sequences were deposited in GenBank under accession numbers MN242989–MN243084.

### 2.4. Amplified Fragment Length Polymorphism (AFLP) Fingerprinting

The genetic diversity of the *C. auris* isolates was assessed by AFLP fingerprinting as described elsewhere [53]. The selective primers used in the current study were HpyCH4IV with one selective residue (5′-FLU-GTAGACTGCGTACCCGTC-3′) and the MseI primer with four selective residues (5′-GATGAGTCCTGACTAATGAT-3′). PCR products were purified and diluted 10× in ddH_2_O. One microliter of diluted PCR product was added to a mixture of 8.9 µL ddH_2_O and 0.1 µL Orange 600 internal size marker (Nimagen, Nijmegen, The Netherlands) followed by 1 min heating at 100 °C, and thereafter directly analyzed on an ABI3730xL Genetic Analyzer (Applied Biosystems). Raw data were imported into BioNumerics v7.6 (Applied Maths) and automatically processed. The assignment of the internal size standard was visually checked and manually corrected if needed. A UPGMA dendrogram was generated with BioloMICS v12 (BioAware).

### 2.5. MALDI-TOF Mass Spectrum Analysis

Overnight cultured *C. auris* isolates grown on SDA were used for spectra acquisition. Full extraction was performed according to the MALDI Biotyper standard protocol as described elsewhere [54,55,56]. Briefly, from each isolate, biomass was taken with a 10 µL inoculation loop and suspended in 500 µL distilled water and centrifuged 3 min at 14,000× *g*, the supernatant was discarded, and 1 mL of 70% ethanol was added. The suspension was homogenized and then centrifuged at 3 min at 14,000× *g*. Following this, based on the pellet size, equal amounts of 70% formic acid and 100% acetonitrile were added. After centrifugation for 5 min at 14,000× *g*, 1 μL of supernatant was spotted eight times onto a polished steel target plate (Bruker Daltonik) and overlaid with 1 µL MALDI matrix (10 mg/mL of α-cyano-4-hydroxy-cinnamic acid (α-HCCA) in 50% acetonitrile–2.5% trifluoroacetic acid; Bruker Daltonik) [57]. MALDI-TOF MS spectra were acquired with a Microflex LT/SH mass spectrometer (Bruker Daltonik) calibrated with the Bruker Bacterial Test Standard in the mass range between 2 and 20 kDa [25]. Up to 24 raw spectra were analyzed by the MALDI Biotyper Compass Explorer 4.1 software (Bruker Daltonik) to generate the reference spectra (MSP—Main Spectra Projection) and a UPGMA dendrogram was created with BioloMICS v12 (BioAware).

### 2.6. IR Biotyper Spectrum Acquisition and Analysis

Overnight cultures of *C. auris* were cultivated on SDA medium. The biomass of two times 10 µL inoculation loops was transferred into 70 µL of 70% (*v/v*) ethanol in 1.5 mL tubes. The tubes were equipped with metal rods to allow for better homogenization (Bruker Daltonik). The suspension was homogenized by 5 min vortexing; then, 70 µL of deionized water was added followed by 5 min additional shaking. Fifteen microliters of the suspension was spotted in triplicate on a silicon sample plate (Bruker Daltonik) and dried at 37 °C. The experiment was repeated on three different days to cover cultural variance. Spectra were acquired using the IR Biotyper system v2.1 (Bruker Daltonik) with the following default analysis settings: 32 scans per sample, 10 kHz scan speed, 6 cm^−1^ resolution, Blackman–Harris 3-term apodization, and zero filling 4. Spectra with an absorption < 0.4 or > 2, a signal/noise ratio < 40 in the carbohydrate range, and/or fringes > 10^−4^ were not considered for further analysis as these will not be in agreement to already defined factors to provide the default quality criteria, and may have led to either failed or wrong outcomes [32]. The original absorption spectra consisting of 3629 data points over the range from 4000 to 500 cm^−1^ were processed as follows: the second derivative was taken over nine data points, cut to 1300–800 cm^−1^, and vector-normalized. For each of the isolates, the arithmetic mean of qualified spectra was calculated, so that each mean spectrum was composed of 3–12 single spectra. A UPGMA dendrogram was generated with BioloMICS v12 (BioAware).

### 2.7. Comparison of Clustering Concordance

Pearson correlation (Mantel test) between the different typing methods was calculated using BioloMICS v12 (BioAware) [58].

## 3. Results

### 3.1. Comparison of the Five Typing Methods

**Figure 1:**
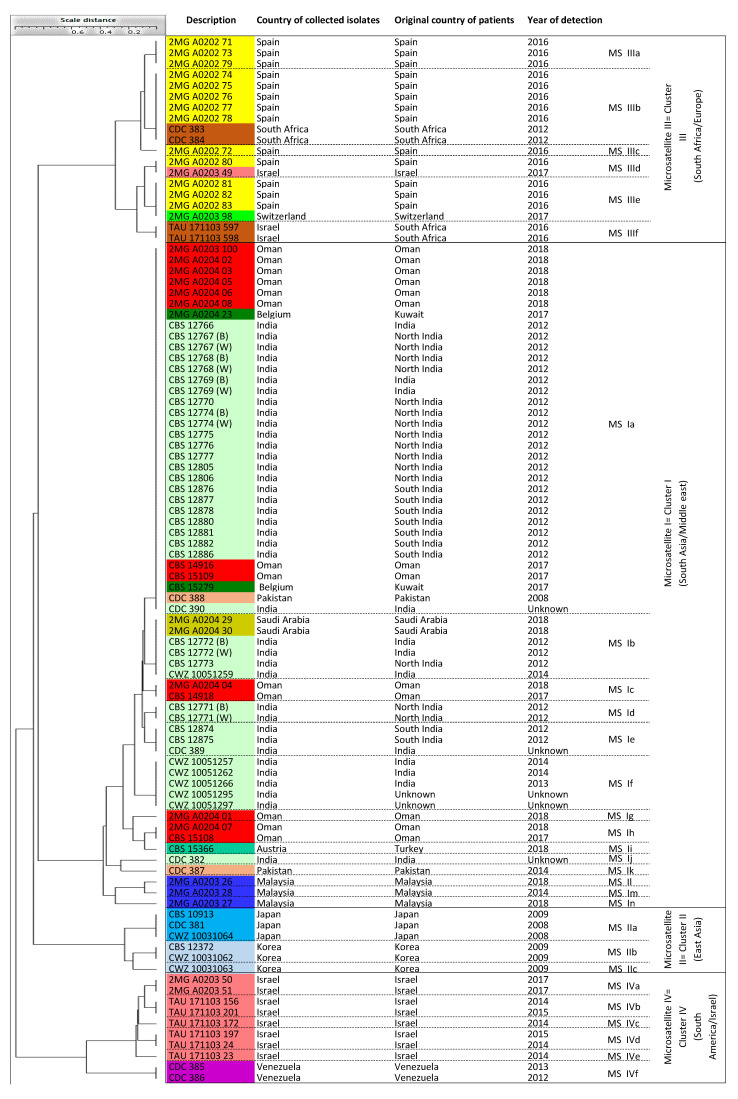
A UPGMA dendrogram was generated with BioloMICS v12 based on microsatellite analysis. Four main geographically linked clusters, namely South Asia/Middle East, East Asia, South Africa/Europe, and South America/Israel were created using 96 isolates. Microsatellite abbreviation (MS) was used for sub-clusters. The color coding used for this dendrogram was the same as follows: Austria (turquoise), Belgium (dark green), Japan (blue), India (light green), Israel (rose), Korea (light blue), Malaysia (dark blue), Oman (red), Pakistan (light pink), Saudi Arabia (chartreuse), Spain (yellow), South Africa (brown), Switzerland (green), and Venezuela (purple).

Five UPGMA-based dendrograms were obtained from the calculated distance matrices. Cophenetic (Mantel test) clustering [59] allowed for a comparison between the distance matrices underlying the respective clustering analyses. A few isolates used in the current study were previously analyzed by whole genome sequence (WGS) analysis and/or microsatellite analysis [19,39]. Comparison of data acquired for those isolates in this study and previous ones showed good agreement between the microsatellite data of the respective analyses (Figure 1, Table 1) [19]. Only the isolate from Israel [19,44], that was previously classified by microsatellite typing in clade III, grouped into cluster IV in the current study.

For the comparison between the five typing methods addressed here, we considered the microsatellite assay as the reference method for *C. auris* typing. As indicated in the analysis of the single typing approaches (see below), some yielded similar clustering patterns to the microsatellite analysis, whereas others differed widely. In general, cophenetic correlation values were low indicating poor concordance between the methods investigated. Using cophenetic clustering analysis, microsatellite typing showed the highest agreement with ITS sequencing showing a correlation of 0.45, followed by results from the IR Biotyper with a score of 0.33 (Table 2). The cophenetic correlation between ITS sequence-based typing and results from MALDI-TOF MS was only 0.29. Even lower agreement was observed between microsatellite typing and MALDI-TOF MS with a score of 0.21, and with AFLP with a score of 0.22 (Table 2). The AFLP analysis showed very low scores with typing results obtained from ITS sequence, MALDI-TOF MS, and IR Biotyper analyses, indicating lack of concordance. There was no agreement between MALDI-TOF MS and IR Biotyper as these methods had a similarity score of −0.011 (Table 2).

Among the 96 tested *C. auris* isolates, isolate number CBS 10913 (Japan) is the type-strain and this strain was included in this study with two other IDs, namely CDC 381 and CWZ 10031064. Additionally, a Korean isolate, namely CBS 12372 (KCTC 17809), was present as duplicate CWZ 10031062. These isolates provided us the capability to test the technical reproducibility of the five typing methods used here. According to the created clusters and sub-clusters, the Japanese triplicate and Korean duplicate isolates correctly grouped into the South East cluster by the microsatellite assay and ITS sequencing. The IR Biotyper typing method classified the triplicated Japanese type-strain into one sub-cluster, whereas the Korean isolates were divided into two sub-clusters. AFLP fingerprinting and MALDI-TOF MS analysis grouped the respective isolates into different clusters and sub-clusters. Furthermore, 10 isolates belonged to the South America cluster and were grouped together with all methods, except MALDI-TOF MS. This method separated one isolate from Venezuela (CDC 386) out of the remaining nine isolates and placed it in cluster MALDI II with isolates from South Asia, East Asia, and South Africa.

### 3.2. Microsatellite Typing

The microsatellite typing analysis showed four main clusters labeled Microsatellite I-IV (MS I-IV) (Figure 1). Sixty-one isolates (63.5%) clustered in MS I, and included isolates of patients originating from Austria (a Turkish patient), Belgium (Kuwaiti patients), India, Malaysia, Oman, Pakistan, and Saudi Arabia. The East Asia group (cluster II = MS II) included six isolates (6.25%) from Japan and Korea. Cluster MS III had 19 isolates (19.8%), was the second largest group, and contained isolates from South Africa and Spain, but also with two isolates from Israel and Switzerland. Finally, cluster MS IV included 10 isolates (10.4%) from Israel (*n* = 8) and Venezuela (*n* = 2). Cluster MS I contained six sub-clusters with strains showing the same microsatellite patterns (Table 1). Sub-cluster MS Ia contained 34 isolates originating from India and Pakistan, but also from the Middle East region. This was also true for sub-cluster MS Ib with six isolates, sub-cluster MS Ie with three isolates, and sub-cluster MS If with five isolates. Remarkably, three isolates from Malaysia were genetically distinct and clustered basally in MS I (MS Il, Im, In). In MS II, the three Japanese isolates were identical (sub-cluster MS IIa), but distinct from the three South Korean isolates (sub-clusters MS IIb and IIc). The duplicated Korean isolates, CBS 12372 = CWZ 10031062, were categorized together in sub-cluster MS IIb. Cluster MS III contained six sub-clusters. Sub-cluster MS IIIb had seven isolates from Spain and South Africa, MS IIIe included four isolates from Spain and Switzerland, MS IIIa had three isolates from Spain, MS IIId had two isolates from Israel and Spain, and MS IIIf contained two isolates from Israel that originated from patients that came from South Africa. Finally, MS IIIc contained only a single Spanish isolate. Cluster MS IV showed six sub-clusters that all had only one or two isolates. Note that the eight Israeli isolates belonging to five sub-clusters of MS IV were genetically distinct from those belonging to clusters MS IIId and MS IIIf. The two isolates from Venezuela had the same microsatellite pattern and were distinct from the Israeli isolates in cluster MS IV. To conclude, three of the four main clusters contained isolates from patients originating from diverse continents (Table 1).

### 3.3. ITS Sequence-Based Typing

**Figure 2:**
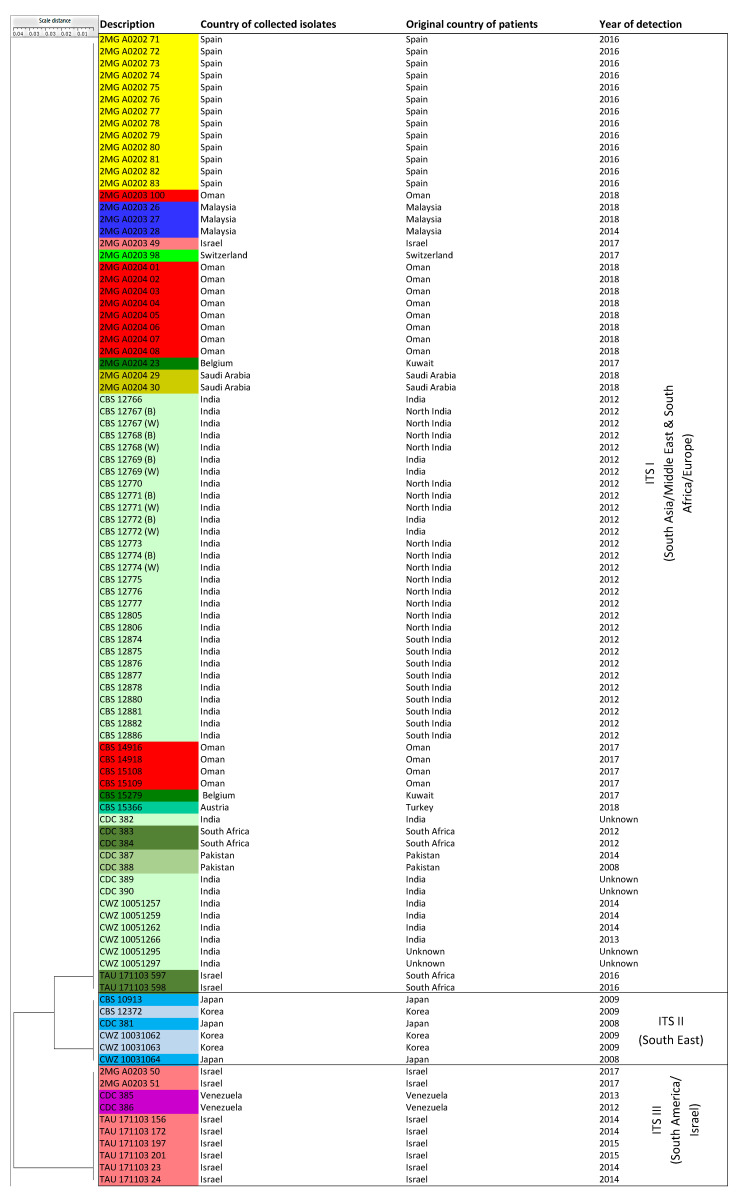
A UPGMA dendrogram generated with BioloMICS v12 made three clusters based on ITS sequences. ITS I included isolates from South Asia/Middle East and South Africa/Europe, ITS II and III included isolates from East Asia and South America/Israel, respectively. The color coding used for this dendrogram was the same as Figure 1.

A UPGMA analysis using the Pearson correlation algorithm divided the 96 *C. auris* isolates into three clusters, ITS I-III. Eighty isolates (83.3%) including Austria (from a Turkish patient), Belgium (Kuwaiti patients), India, Israel, Malaysia, Oman, Pakistan, Saudi Arabia, South Africa, Spain, and Switzerland had identical ITS sequences. The cluster, ITS I, contained representatives of clusters MS I and MS III as revealed by the microsatellite typing analysis. The second cluster ITS II was fully concordant with cluster MS II, as revealed by microsatellite typing, and had six isolates (6.25%) from Japan and Korea. The remaining group, cluster ITS IV, had the same 10 isolates (10.42%) from Israel and Venezuela as the microsatellite typing identified cluster MS IV (Figure 2, Table 1).

### 3.4. AFLP Fingerprinting

**Figure 3:**
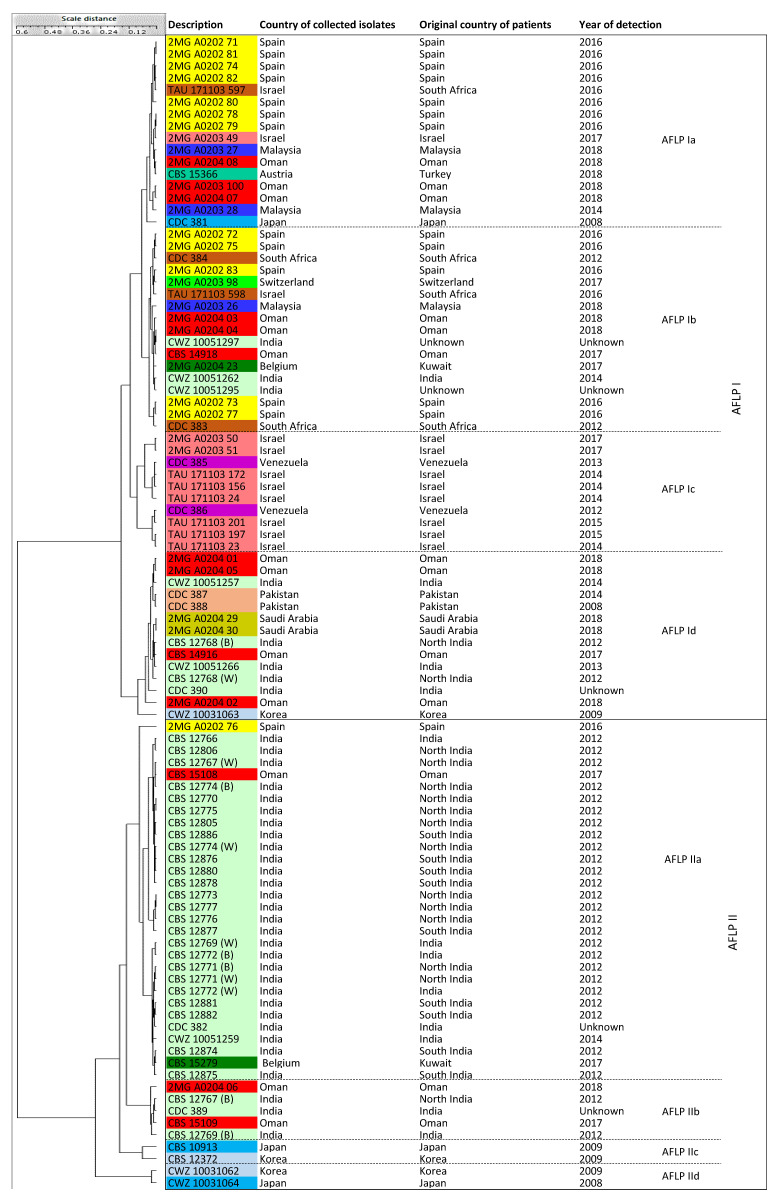
A UPGMA dendrogram was generated with BioloMICS v12 based on AFLP genotyping results. Two main clusters, namely, AFLP I and AFLP II, were created and each was divided into four sub-clusters, AFLP Ia-Id and AFLP IIa-IId. The color coding used for this dendrogram was the same as Figure 1.

AFLP fingerprinting clustered the 96 *C. auris* isolates into two main clusters (Figure 3), and each of these two clusters were divided into four sub-clusters (AFLP Ia-d and AFLP IIa-d). Isolates from Austria (from a Turkish patient), Belgium (Kuwaiti patients), India, Israel, Korea, Malaysia, Oman, Pakistan, and Saudi Arabia belonged to clusters AFLP Ia, Ib, Id, IIa, and IIb. Similarly, isolates from Spain and South Africa belonged to clusters AFLP Ia, Ib, and IIa. Two Israeli isolates with the same microsatellite typing pattern MS IIIf, namely, TAU 171103597 and TAU 171103598 that both came from patients originating from South Africa, clustered in the clusters AFLP Ia and Ib. Cluster MS IV remained intact in the AFLP analysis as cluster AFLP Ic. Indian isolates belonged to cluster MS I and showed quite some genetic divergence by AFLP fingerprinting, indicating that they are not genetically homogeneous. For instance, isolates from microsatellite cluster MS Ia belonged to clusters AFLP Id, IIa, and IIb (Figure 1 and Figure 3; Table 1). Isolates from MS If clustered into AFLP clusters Ib and Id. The Japanese duplicated type-strains (CBS 10913 and CWZ 10031064) were located in cluster AFLP II (sub-clusters IIc and IId, respectively). In contrast, the third strain from this type strain, namely CDC 381, grouped in cluster AFLP Ia that was distinct from sub-clusters IIc and IId. The duplicated Korean isolates, CBS 12372 = CWZ 10031062, were both in cluster AFLP II, but in different sub-clusters IIc and IId, respectively. Another Korean isolate, CWZ 10031063, was classified into AFLP Id.

### 3.5. MALDI-TOF MS

**Figure 4:**
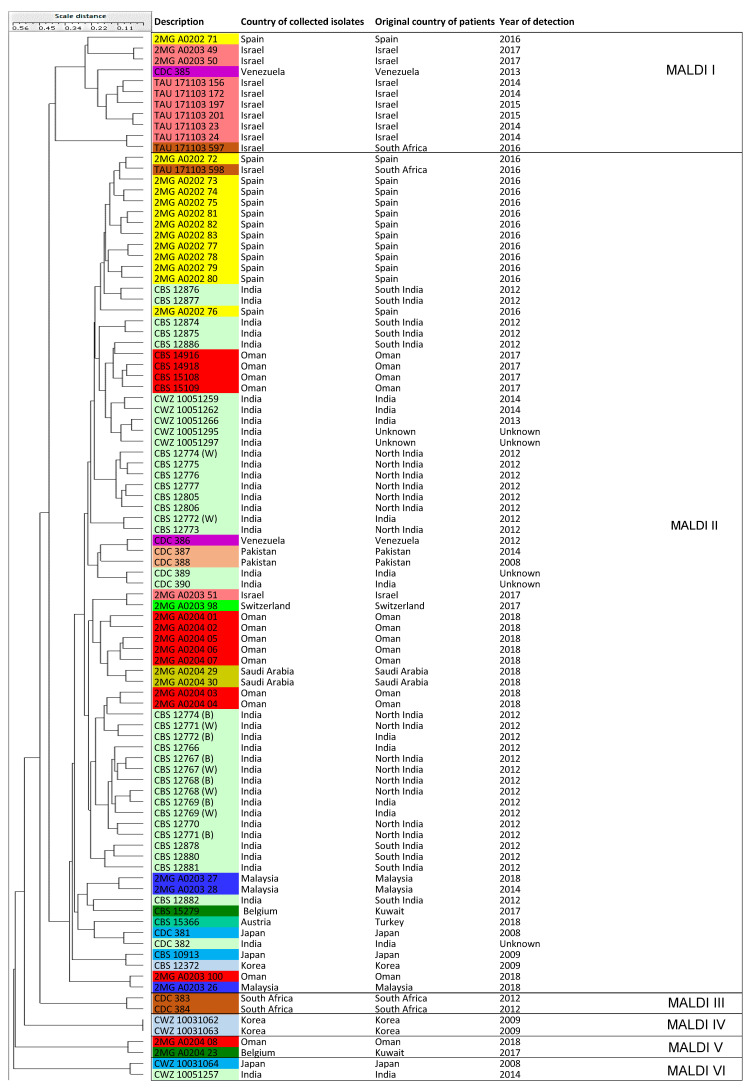
A UPGMA dendrogram was generated with BioloMICS v12 for 96 *C. auris* isolates by MALDI-TOF MS. Two main clusters were made, namely, MALDI I and MALDI II, with eight isolates forming basal lineages without assignment to a cluster. The color coding used for this dendrogram was the same as Figure 1.

A dendrogram was generated based on the MALDI-TOF MS MSPs, yielding in two distinct clusters, MALDI I and MALDI II, with eight isolates forming basal lineages without assignment to a cluster. Cluster MALDI I contained isolates from MS III and MS IV, but cluster MALDI II had isolates of all four major microsatellite clusters (MS I-IV) that were previously recognized. The eight basal isolates belonged to clusters MS I, MS II, and MS III. Given the considerable proteomic variation, we refrained from assigning further sub-clusters (Figure 4). Two duplicates of the Japanese type-strain, namely, CBS 10913 and CDC 381, grouped in cluster MALDI II together with a Korean isolate (CBS 12372), while the third type-strain, CWZ 10031064, formed a small group together with the Indian isolate CWZ 10051257. Two other Korean isolates grouped together as sub-cluster MALDI IV.

### 3.6. IR Biotyper

**Figure 5:**
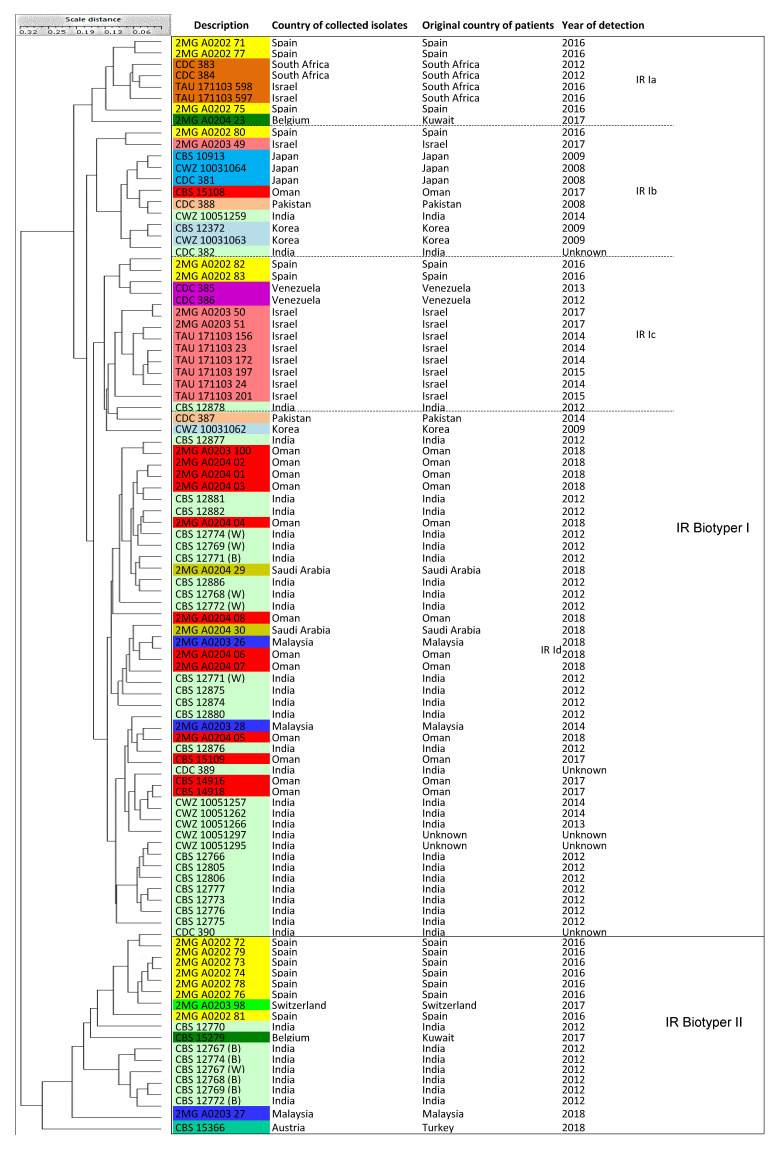
A UPGMA dendrogram generated with BioloMICS v12 of spectra generated by IR Biotyper. Two major clusters were created, namely, IR I and IR II, and among them, cluster IR I was divided into four sub-clusters, IR Ia-Id. IR Biotyper abbreviation (IR) was used for sub-clusters. The colur coding used for this dendrogram was the same as Figure 1.

In total, 860 IR spectra of 96 *C. auris* isolates were measured. Figure 5 shows that two major clusters can be recognized, namely IR I with 78 isolates (81.25%) and IR II with 18 isolates (18.75%). Similar to the MALDI-TOF MS-based clustering, no concordance was observed between the IR Biotyper clustering and the previously assigned main clusters based on microsatellite typing (Figure 5, Table 1). Isolates of cluster IR I were classified to four sub-clusters (IR Ia-Id) that each agreed with one of four major MS clusters, whereas IR II contained representatives of clusters MS I and MS III. Given the high level of diversity within genotype IR II, we refrained from assigning further subgroups. IR Biotyper clustered all six isolates from East Asia into the IR Ib cluster, excluding the Korean isolate CWZ 10031062 that occurred in the sub-cluster IR Id.

## 4. Discussion

This study was undertaken to evaluate the applicability of IR Biotyper as a biochemical typing method to type *C. auris* isolates and compare the outcome with results obtained by MALDI-TOF MS and molecular typing methods. The main goal for the development of the IR Biotyper method was to establish a rapid typing method to investigate the relatedness between isolates involved in nosocomial outbreaks [29,36]. In this study, the five typing approaches, IR Biotyper, MALDI-TOF MS, ITS sequencing, AFLP fingerprinting, and microsatellite typing, did not show concordance.

Microsatellite typing is a rapid molecular typing method used for the analysis of genetic variation in and between fungal populations [21,60] and has been broadly used for typing *Aspergillus flavus*, *Aspergillus fumigatus*, and *Candida* species, such as *Candida albicans*, *Candida glabrata*, *Candida parapsilosis*, and *Candida tropicalis*, as well as to investigate three decades of *Cryptococcus neoformans* epidemiology in the Netherlands [20,21,61,62,63,64]. In this study, the results obtained by microsatellite typing in which four clusters, MS I-IV, were recognized, had the best concordance with typing studies based on WGS analysis [17,39], gene sequencing [18], and a previous microsatellite typing study [19]. WGS, as the presumed gold standard, provided background information about the previously geographically defined four clades I-IV. All four WGS CDC reference strains were correctly classified using the microsatellite assay into their respective clusters. The only exception related to cluster MS III that included one Israeli isolate (2MG A0203 49) and one isolate from Switzerland (2MG A0203 98). Since all Israeli isolates were grouped in cluster IV (=South America) with isolates from Venezuela, the history of single Israeli isolate (2MG A0203 49) located in cluster III was investigated. The Israeli patient had a motor vehicle accident in South Africa after which he was repatriated to Israel while infected with *C. auris* [44]. The isolate from Switzerland (2MG A0203 98) has not been used in other studies and we could not compare the results; however, the Swiss patient was hospitalized in Geneva after a vacation in northwestern Spain where she was admitted to a local hospital [50]. Therefore, the patient’s travel history could explain some peculiar results we obtained in our typing studies. Furthermore, the data in this study largely corroborated results from a recent microsatellite typing study that included 444 *C. auris* isolates (Figure 1, Supplementary Table S1) [19]. We conclude that microsatellite analysis is a useful and easy-to-use typing method with high reproducibility and portability to understand geographical patterns of *C. auris* populations, and has lower costs compared with WGS [65].

ITS sequencing largely corroborated the clusters found by microsatellite analysis, except for isolates from South Africa and Spain (MS III) that grouped together with isolates from South Asia (MS I) and that clustered differently with microsatellite typing. Thus, ITS sequence-based typing has less resolution than microsatellite analysis. The CDC *C. auris* reference strains, CDC 388 (Pakistan), CDC 381 (Japan), and CDC 385 (Venezuela), were correctly classified into ITS I, ITS II, and ITS IV, respectively, while isolate CDC 383 (South Africa) was grouped incorrectly into ITS I.

AFLP fingerprinting has remained a method with broad applications in outbreak investigations, microbial clustering studies, and typing [18,66,67,68,69]. This method has high robustness to discriminate between isolates and species; additionally, compared to other typing methods, such as microsatellite typing and WGS analysis, AFLP fingerprinting is cheaper and requires less time. However, its reproducibility remains a problem as data from two experiments [17,19,69,70,71] cannot be combined. In the AFLP study of Chowdhary et al. (2013), a small sample size (30 isolates) was used to classify *C. auris* isolates according to the respective geographical origin. The isolates from India clustered together, and the isolates from Japan and Korea created another cluster. In their study, AFLP method could successfully classify isolates into two different clusters based on the isolates’ geographical origins [72]. Next, Prakash et al. (2016) performed a study using a larger sample size of 104 isolates. They focused on isolates from India, Venezuela, and South Africa plus two control isolates from Japan and Korea [18]. Their AFLP analysis classified Indian and South American isolates into two major clusters, and the South African isolates were randomly distributed [18]. In our study, AFLP categorized isolates into two main clusters (AFLP I and AFLP II) and each one included four sub-clusters. Thus, previously recognized clusters obtained by microsatellite typing and ITS sequencing did not, to a large extent, corroborate with the obtained AFLP clustering. A comparison of AFLP fingerprinting with microsatellite typing indicated that the isolates from South Asia and East Asia were apparently randomly divided into both the main AFLP clusters, while the isolates from the presumed South African (South Africa and Spain) and South American (Venezuela and Israel) clades were located in cluster AFLP I. The reference isolates CDC 381 (Japan), CDC 383 (South Africa), CDC 385 (Venezuela), and CDC 388 (Pakistan) were distributed into cluster AFLP I (sub-clusters AFLP Ia, AFLP Ib, AFLP Ic, and AFLP Id, respectively).

MALDI-TOF MS is a protein-based method that has been used for *Candida* typing in several studies [31,66,73,74]. In the current study, six MALDI-TOF MS clusters were generated and when compared with microsatellite typing, poor agreement was observed between both methods with a correlation of only 0.21. MALDI-TOF MS differentiates between isolates of the same species based on the diversity of the proteome and this may not always provide a similar result of the intrinsic genetic variation of microorganisms due to post-translational modification (PTM) and other experimental variables that influence the protein structure and, consequently, the spectra. Acquired MSPs could be variable due to technical issues, such as accuracy in protein extraction and pipetting samples onto a polished steel target plate. Alternatively, MSP differences could be problematic because they were calculated based on standard MSP settings, which are optimized for species differentiation, not for cluster analysis. Thus, MALDI-TOF MS may not be useful to differentiate between isolates belonging to the same species, as relatively few peaks with differences are generated. Indeed, the gross similarity between the spectra may obviate its utility for intra-species typing [18,73,75,76]. The MSP-based typing method generates a dendrogram based on log(scores) obtained for each isolate individually and compares them with scores obtained for the other isolates. If there is no significant variation between the log(scores), which might be the case for clonally related isolates, the MSP-based dendrogram analysis may not produce an accurate result [18,75]. Moreover, the MSP-based dendrogram should always contain an outlier isolate, because without such an outlier the dendrogram may result in over-interpretation. For instance, using some *Candida albicans* and *Candida glabrata* isolates as outliers might affect the acquired results differently. The lack of an outlier species in this study may explain why the MALDI-TOF MS-based dendrogram differed widely from the clustering obtained by microsatellite analysis. The use of BioloMICS v12 (BioAware) to generate a UPGMA dendrogram might not have been the optimal analysis method for the acquired MSPs generated by MALDI-TOF MS. Other available analytical methods, such as BioNumerics, and a comparison between dendrograms created by these different approaches may improve the analytical outcomes generated as MSP-based dendrograms. Four MALDI-TOF MS clusters, MALDI III-VI, each containing only two isolates, were generated that occurred distantly from the main MALDI-TOF MS clusters (MALDI I and MALDI II). The reference strain CDC 385 (Venezuela) clustered in MALDI I that included isolates from Israel and Venezuela (=cluster MS IV = South American clade); isolate CDC 383 (South Africa) created a small group with another isolate from the same region (CDC 384) that belonged to the South Africa cluster; isolates CDC 381 (Japan) and CDC 388 (Pakistan) were located in the MALDI II cluster that included isolates from MS I, MS II, MS III, and MS IV. Overall, the MALDI Biotyper provides excellent performance for species identification; however, it is not useful for typing different *C. auris* strains when compared to the microsatellite typing assay.

The acquisition of IR Biotyper spectra depends highly on the conditions used during culturing of the yeasts [32,77]. The method is rapid, providing final results in 1–2 h per sample and is easy to use without any prior knowledge of FTIR technology. This method has been developed with a possibility to change different parameters during the analysis to obtain different clustering patterns. Using the IR Biotyper method, each isolate was measured nine times and the final result was normalized by merging the nine acquired spectra. Consequently, a single misclassification due to the impact of one of the variable factors should not affect the clustering. Moreover, by measuring every isolate nine times in this manner, the reproducibility of the IR Biotyper method increases in microbial typing according to the manufacturer’s recommendations. In this study, IR Biotyper analysis created two main clusters, namely IR I and IR II. Comparing IR Biotyper and microsatellite typing results revealed an agreement with a correlation score of 0.33, which ranked IR Biotyper as showing the second-highest agreement with microsatellite typing after ITS sequencing (Table 2). Two Israeli isolates that originated from South Africa (TAU 171103 597 and TAU 171103 598) were placed in sub-cluster IR Ia (cluster III = South Africa), which was in agreement with the microsatellite typing analysis, and supported the findings by Belkin et al. (2017) [44] who reported that the Israeli *C. auris* isolates originally came from South Africa. Isolate numbers CDC 381 (Japan) and CDC 388 (Pakistan) grouped in the major cluster IR I (sub-cluster IR Ib) with isolates from India, Israel, Oman, and Spain. CDC 383 (South Africa) and CDC 385 (Venezuela) grouped in sub-clusters IR Ia and IR Ic and belonged to cluster MS III (South Africa) and MS IV (South America), respectively. Thus, more effort is required to evaluate and optimize several parameters in measuring and analyzing data obtained by FTIR spectroscopy before this method can be used to investigate intra-species types in hospital outbreaks caused by *C. auris* and, likely, isolates of other yeast species.

A further analysis was performed to evaluate the reproducibility of the five typing methods investigated in this study using triplicated *C. auris* Japanese type-strain, and duplicate Korean isolates. The observed result after microsatellite and ITS typing showed the high technical reproducibility of both methods. The IR Biotyper method indicated reliable reproducibility by clustering all Japanese type-strains into one sub-cluster. In contrast, AFLP fingerprinting and MALDI-TOF MS analysis placed the triplicated Japanese and duplicate Korean isolates in non-concordant subgroups that referred to their low power of technical reproducibility.

In summary, none of the typing methods explored in this study showed a concordance above 50%. Comparing the results obtained by the microsatellite typing assay, the other methods created different clusters or sub-clusters, including separating or combining isolates from different countries and continents. This observation is difficult to explain, but lack of information about the patient’s background may impact our understanding of the epidemiology of *C. auris* infections. In addition, this result may also be due to intrinsic genetic, biochemical, or phenotypic variations among *C. auris* isolates. Genomic variation can be generated via small local alteration of the nucleotide sequence of the genome, intragenomic restructuring, and the acquisition of genetic material from other *Candida* species or even other microbes to adapt to different environmental conditions as well as the human body [78,79,80]. A recent study based on a WGS analysis showed limited genetic diversity between isolates within each of the four *C. auris* clades, whereas genetic diversity was obvious between the four clades [17]. All analyses in our study, except the results from the microsatellite typing method, showed extensive variation, but no clear clustering according to the previously defined four main clades. As an exception, eight out of ten South American (=clade IV) isolates showed an identical clustering in all five typing methods used in this study that were largely in agreement with results obtained from other typing studies (Supplementary Table S1). Taken together, to obtain a correct insight into the epidemiological distribution of *C. auris*, further analyses based on a larger and blinded sample size and inclusion of various independently working laboratories, using WGS as a reference, are needed to evaluate other less costly methods as explored here.

## Figures and Tables

**Table 1:** A summary of the clusters and sub-clusters created by the five typing methods used in this study.

	Clusters	Sub-Clusters	Number of Isolates (Known Cluster Names Based on Microsatellite Assay and Countries of Origin)
**Microsatellite assay**	Microsatellite I	19 sub-clusters (Ia-n)	61 isolates (Cluster MS I = South Asia/Middle East)
	Microsatellite II	3 sub-clusters (IIa-c)	6 isolates (Cluster MS II = East Asia)
	Microsatellite III	6 sub-clusters (IIIa-f)	19 isolates (Cluster MS III = Israel, South Africa, Spain, Switzerland)
	Microsatellite IV	6 sub-clusters (IVa-b)	10 isolates (Cluster MS IV = Israel, South America)
**ITS sequencing**	ITS I	NA	80 isolates (Cluster MS I and III = South Africa, South Asia/Middle East, Spain, Switzerland)
	ITS II	NA	6 isolates (Cluster MS II = East Asia)
	ITS III	NA	10 isolates (Cluster MS IV = Israel, South America)
**AFLP genotyping assay**	AFLP I	4 sub-clusters (Ia-d)	39 isolates (Cluster MS I, II, III, IV)
	AFLP II	4 sub-clusters (IIa-d)	57 isolates (Cluster MS I, II, III = East Asia, South Asia/Middle East, Spain)
**MALDI-TOF MS**	MALDI-TOF I	NA	11 isolates (Cluster MS III and Cluster IV = Israel, South America, Spain)
	MALDI-TOF II	NA	77 isolates (Cluster MS I, II, III, Cluster IV)
	MALDI-TOF III	NA	2 isolates (Cluster MS III = South Africa)
	MALDI-TOF IV	NA	2 isolates (Cluster MS II = Korea)
	MALDI-TOF V	NA	2 isolates (Cluster MS I = Oman, Kuwait)
	MALDI-TOF VI	NA	2 isolates (Cluster MS I, II = India, Japan)
**IR Biotyper**	IR Biotyper I	4 sub-clusters (Ia-d)	78 isolates (Cluster MS I, II, III, IV)
	IR Biotyper II	NA	18 isolates (Cluster MS I, III = India, Kuwait, Malaysia, Spain, Switzerland, Turkey)

**Table 2:** Correlation between the results obtained with five typing methods used for 96 *Candida auris* isolates.

Method	ITS Sequencing	MALDI-TOF MS	AFLP	IR Biotyper	Microsatellites
**ITS sequencing**		0.285	0.065	−0.010	0.446
**MALDI-TOF MS**	0.285		0.052	−0.011	0.210
**AFLP**	0.065	0.052		0.061	0.215
**IR Biotyper**	−0.010	−0.011	0.061		0.330
**Microsatellites**	0.446	0.210	0.215	0.330	

Data were calculated by Pearson correlation between the methods by using BioloMICS v12 software. Green colors show the highest similarities between two methods, followed by orange (middle agreements) and red (lowest agreements). The highest similarity was observed between microsatellite assay and ITS sequencing followed by microsatellite assay and IR Biotyper typing method. The lowest similarity was obtained between microsatellite and MALDI-TOF MS.

## Data Availability

Sequence data have been submitted to GenBank with the accession numbers MN242989–MN243084. *Candida auris* has been deposited to the CBS culture collection (hosted at the Westerdijk Fungal Biodiversity Institute, Utrecht, The Netherlands).

## Supplementary Materials

The following are available online at https://www.mdpi.com/2309-608X/6/3/146/s1, Table S1: Overview of 96 Candida auris strains used in this study.
