# Supplementary material for: Evaluation of Microsatellite Typing, ITS Sequencing, AFLP Fingerprinting, MALDI-TOF MS, and Fourier-Transform Infrared Spectroscopy Analysis of Candida auris
Source: J Fungi (Basel). 2020 Aug 25;6(3):146. doi: 10.3390/jof6030146 (PMC7576496; doi:10.3390/jof6030146)
Supplement: Supplementary file 1 [file jof-06-00146-s001.pdf]

Supplementary Material

Supplementary Table 1. Overview of 96 *Candida auris* strains used in this study is shown in this table. The only discrepancy between the clusters found by other studies and the microsatellite in this study is highlighted by red color. Isolates marked with purple color belonged to cluster IV (South America clade) have shown identical clustering in all five typing methods used in this study compared to the clusters (=clades) obtained from other studies based-molecular techniques. This shows that these isolates are highly genetically identical, but contrary, there is an obvious genetic diversity between these isolates compared to those from the other three clades.

Isolate number [(a)]	Other isolate numbers	Country	Patients country of origin	Clades (Clusters) according to other studies (WGS, ITS, Microsatellite)[1-3]	Clusters-according to microsatellite typing results in this study	Year of detection (Identification)
CDC 381 [b]	B11220	Japan	Japan	Cluster II (East Asia clade)	Cluster MS II (East Asia)	2008
CDC 382		India	India	Cluster I (South Asia clade)	Cluster MS I (South Asia/Middle East)	Unknown
CDC 383	B11221	South Africa	South Africa	Cluster III (South Africa clade)	Cluster MS III (South Africa/Europe)	2012
CDC 384	B11222	South Africa	South Africa	Cluster III (South Africa clade)	Cluster MS III (South Africa/Europe)	2012
CDC 385	B11243	Venezuela	Venezuela	Cluster IV (South America clade)	Cluster MS IV (South America/Israel)	2013
CDC 386	B11244	Venezuela	Venezuela	Cluster IV (South America clade)	Cluster MS IV (South America/Israel)	2012
CDC 387	B11098	Pakistan	Pakistan	Cluster I (South Asia clade)	Cluster MS I (South Asia/Middle East)	2014
CDC 388	B8441	Pakistan	Pakistan	Cluster I (South Asia clade)	Cluster MS I (South Asia/Middle East)	2008
CDC 389		India	India	Cluster I (South Asia clade)	Cluster MS I (South Asia/Middle East)	Unknown
CDC 390		India	India	Cluster I (South Asia clade)	Cluster MS I (South Asia/Middle East)	Unknown
TAU-171103-23	B11894	Israel	Israel	Cluster IV (South America clade)	Cluster MS IV (South America/Israel)	2014
TAU-171103-24	B11896	Israel	Israel	Cluster IV (South America clade)	Cluster MS IV (South America/Israel)	2014
TAU-171103-156	B11892	Israel	Israel	Cluster IV (South America clade)	Cluster MS IV (South America/Israel)	2014
TAU-171103-172	B11893	Israel	Israel	Cluster IV (South America clade)	Cluster MS IV (South America/Israel)	2014
TAU-171103-197	B11895	Israel	Israel	Cluster IV (South America clade)	Cluster MS IV (South America/Israel)	2015
TAU-171103-201	B11897	Israel	Israel	Cluster IV (South America clade)	Cluster MS IV (South America/Israel)	2015
TAU-171103-597	B11223	Israel	South Africa	Cluster III (South Africa clade)	Cluster MS III (South Africa/Europe)	2016

TAU-171103-598	B11224	Israel	South Africa	Cluster III (South Africa clade)	Cluster MS III (South Africa/Europe)	2016
CWZ-10031062	B11808 (KCTC 17809)	Korea	Korea	Cluster II (East Asia clade)	Cluster MS II (East Asia)	2009
CWZ-10031063	B11809 (KCTC 17810)	Korea	Korea	Cluster II (East Asia clade)	Cluster MS II (East Asia)	2009
CWZ- 10031064 [b]	B11220 (CDC 381)	Japan	Japan	Cluster II (East Asia clade)	Cluster MS II (East Asia)	2008
CWZ-10051257	VPCI 467/P/14	India	India	Cluster I (South Asia clade)	Cluster MS I (South Asia/Middle East)	2014
CWZ-10051259	VPCI 471A/P/14	India	India	Cluster I (South Asia clade)	Cluster MS I (South Asia/Middle East)	2014
CWZ-10051262	VPCI 478/P/14	India	India	Cluster I (South Asia clade)	Cluster MS I (South Asia/Middle East)	2014
CWZ-10051266	VPCI 1133/P/13	India	India	Cluster I (South Asia clade)	Cluster MS I (South Asia/Middle East)	2013
CWZ-10051295		India	Unknown	Cluster I (South Asia clade)	Cluster MS I (South Asia/Middle East)	Unknown
CWZ-10051297		India	Unknown	Cluster I (South Asia clade)	Cluster MS I (South Asia/Middle East)	Unknown
2MG-A0204-23	05-299	Belgium	Kuwait	Cluster I (South Asia/Middle East clade)	Cluster MS I (South Asia/Middle East)	2017
CBS 15279	Repeat of 05-299	Belgium	Kuwait	Cluster I (South Asia/Middle East clade)	Cluster MS I (South Asia/Middle East)	2017
CBS 10913 [b]	JCM 15448	Japan	Japan	Cluster II (East Asia clade)	Cluster MS II (East Asia)	2009
CBS 12372	KCTC 17809	Korea	Korea	Cluster II (East Asia clade)	Cluster MS II (East Asia)	2009
CBS 12766	VPCI669/P/12	India	India	Cluster I (South Asia clade)	Cluster MS I (South Asia/Middle East)	2012
CBS 12770	VPCI673/P/12	India	India	Cluster I (South Asia clade)	Cluster MS I (South Asia/Middle East)	2012
CBS 12773	VPCI 677/P/12	India	India	Cluster I (South Asia clade)	Cluster MS I (South Asia/Middle East)	2012
CBS 12775	VPCI 709/P/12	India	India	Cluster I (South Asia clade)	Cluster MS I (South Asia/Middle East)	2012
CBS 12776	VPCI711/P/12	India	India	Cluster I (South Asia clade)	Cluster MS I (South Asia/Middle East)	2012
CBS 12777	VPCI 712/P/12	India	India	Cluster I (South Asia clade)	Cluster MS I (South Asia/Middle East)	2012
CBS 12805	VPCI 683/p/12	India	India	Cluster I (South Asia clade)	Cluster MS I (South Asia/Middle East)	2012
CBS 12806	VPCI 692/p/12	India	India	Cluster I (South Asia clade)	Cluster MS I (South Asia/Middle East)	2012
CBS 12876	VPCI473	India	India	Cluster I (South Asia clade)	Cluster MS I (South Asia/Middle East)	2012
CBS 12877	VPCI474	India	India	Cluster I (South Asia clade)	Cluster MS I (South Asia/Middle East)	2012
CBS 14916		Oman	Oman	Cluster I (South Asia/Middle East clade)	Cluster MS I (South Asia/Middle East)	2017
CBS 14918		Oman	Oman	Cluster I (South Asia/Middle East clade)	Cluster MS I (South Asia/Middle East)	2017
CBS 15108	317062126	Oman	Oman	Cluster I (South Asia/Middle East clade)	Cluster MS I (South Asia/Middle East)	2017
CBS 15109	317052804	Oman	Oman	Cluster I (South Asia/Middle East clade)	Cluster MS I (South Asia/Middle East)	2017
CBS 12767 (B)	VPCI670/P/12	India	India	Cluster I (South Asia clade)	Cluster MS I (South Asia/Middle East)	2012
CBS 12767 (W)	VPCI670/P/12	India	India	Cluster I (South Asia clade)	Cluster MS I (South Asia/Middle East)	2012
CBS 12768 (B)	VPCI671/P/12	India	India	Cluster I (South Asia clade)	Cluster MS I (South Asia/Middle East)	2012
CBS 12768 (W)	VPCI671/P/12	India	India	Cluster I (South Asia clade)	Cluster MS I (South Asia/Middle East)	2012

CBS 12769 (B)	VPCI672/P/12	India	India	Cluster I (South Asia clade)	Cluster MS I (South Asia/Middle East)	2012
CBS 12769 (W)	VPCI672/P/12	India	India	Cluster I (South Asia clade)	Cluster MS I (South Asia/Middle East)	2012
CBS 12771 (B)	VPCI674/P/12	India	India	Cluster I (South Asia clade)	Cluster MS I (South Asia/Middle East)	2012
CBS 12771 (W)	VPCI674/P/12	India	India	Cluster I (South Asia clade)	Cluster MS I (South Asia/Middle East)	2012
CBS 12772 (B)	VPCI676/P/12	India	India	Cluster I (South Asia clade)	Cluster MS I (South Asia/Middle East)	2012
CBS 12772 (W)	VPCI676/P/12	India	India	Cluster I (South Asia clade)	Cluster MS I (South Asia/Middle East)	2012
CBS 12774 (B)	VPCI708/P/12	India	India	Cluster I (South Asia clade)	Cluster MS I (South Asia/Middle East)	2012
CBS 12774 (W)	VPCI708/P/12	India	India	Cluster I (South Asia clade)	Cluster MS I (South Asia/Middle East)	2012
CBS 15366		Austria	Turkey	Cluster I (South Asia clade)	Cluster MS I (South Asia/Middle East)	2018
CBS 12874	VPCI471	India	India	Cluster I (South Asia clade)	Cluster MS I (South Asia/Middle East)	2012
CBS 12875	VPCI472	India	India	Cluster I (South Asia clade)	Cluster MS I (South Asia/Middle East)	2012
CBS 12878	VPCI475	India	India	Cluster I (South Asia clade)	Cluster MS I (South Asia/Middle East)	2012
CBS 12880	VPCI477	India	India	Cluster I (South Asia clade)	Cluster MS I (South Asia/Middle East)	2012
CBS 12881	VPCI478	India	India	Cluster I (South Asia clade)	Cluster MS I (South Asia/Middle East)	2012
CBS 12882	VPCI479	India	India	Cluster I (South Asia clade)	Cluster MS I (South Asia/Middle East)	2012
CBS 12886	VPCI483	India	India	Cluster I (South Asia clade)	Cluster MS I (South Asia/Middle East)	2012
2MG-A0202-071		Spain	Spain	Cluster III (South Africa clade)	Cluster MS III (South Africa/Europe)	2016
2MG-A0202-072		Spain	Spain	Cluster III (South Africa clade)	Cluster MS III (South Africa/Europe)	2016
2MG-A0202-073		Spain	Spain	Cluster III (South Africa clade)	Cluster MS III (South Africa/Europe)	2016
2MG-A0202-074		Spain	Spain	Cluster III (South Africa clade)	Cluster MS III (South Africa/Europe)	2016
2MG-A0202-075		Spain	Spain	Cluster III (South Africa clade)	Cluster MS III (South Africa/Europe)	2016
2MG-A0202-076		Spain	Spain	Cluster III (South Africa clade)	Cluster MS III (South Africa/Europe)	2016
2MG-A0202-077		Spain	Spain	Cluster III (South Africa clade)	Cluster MS III (South Africa/Europe)	2016
2MG-A0202-078		Spain	Spain	Cluster III (South Africa clade)	Cluster MS III (South Africa/Europe)	2016
2MG-A0202-079		Spain	Spain	Cluster III (South Africa clade)	Cluster MS III (South Africa/Europe)	2016
2MG-A0202-080		Spain	Spain	Cluster III (South Africa clade)	Cluster MS III (South Africa/Europe)	2016
2MG-A0202-081		Spain	Spain	Cluster III (South Africa clade)	Cluster MS III (South Africa/Europe)	2016
2MG-A0202-082		Spain	Spain	Cluster III (South Africa clade)	Cluster MS III (South Africa/Europe)	2016
2MG-A0202-083		Spain	Spain	Cluster III (South Africa clade)	Cluster MS III (South Africa/Europe)	2016
2MG-A0203-026	UZ495-18	Malaysia	Malaysia	Cluster I (South Asia clade)	Cluster MS I (South Asia/Middle East)	2018
2MG-A0203-027	UZ681-18	Malaysia	Malaysia	Cluster I (South Asia clade)	Cluster MS I (South Asia/Middle East)	2018
2MG-A0203-028	UZ1447-14	Malaysia	Malaysia	Cluster I (South Asia clade)	Cluster MS I (South Asia/Middle East)	2014

2MG-A0203-049		Israel	Israel	Cluster IV (South America clade)	Cluster MS III (South Africa/Europe)	2017
2MG-A0203-050		Israel	Israel	Cluster IV (South America clade)	Cluster MS IV (South America/Israel)	2017
2MG-A0203-051		Israel	Israel	Cluster IV (South America clade)	Cluster MS IV (South America/Israel)	2017
2MG-A0203-098	SMW.2018.14622	Switzerland	Switzerland	NA	Cluster MS III (South America/Israel)	2017
2MG-A0203-100		Oman	Oman	Cluster I (South Asia/Middle East clade)	Cluster I (South Asia/Middle East)	2018
2MG-A0204-001		Oman	Oman	Cluster I (South Asia/Middle East clade)	Cluster I (South Asia/Middle East)	2018
2MG-A0204-002		Oman	Oman	Cluster I (South Asia/Middle East clade)	Cluster I (South Asia/Middle East)	2018
2MG-A0204-003		Oman	Oman	Cluster I (South Asia/Middle East clade)	Cluster I (South Asia/Middle East)	2018
2MG-A0204-004		Oman	Oman	Cluster I (South Asia/Middle East clade)	Cluster I (South Asia/Middle East)	2018
2MG-A0204-005		Oman	Oman	Cluster I (South Asia/Middle East clade)	Cluster I (South Asia/Middle East)	2018
2MG-A0204-006		Oman	Oman	Cluster I (South Asia/Middle East clade)	Cluster I (South Asia/Middle East)	2018
2MG-A0204-007		Oman	Oman	Cluster I (South Asia/Middle East clade)	Cluster I (South Asia/Middle East)	2018
2MG-A0204-008		Oman	Oman	Cluster I (South Asia/Middle East clade)	Cluster I (South Asia/Middle East)	2018
2MG-A0204-029		Saudi Arabia	Saudi Arabia	Cluster I (South Asia/Middle East clade)	Cluster I (South Asia/Middle East)	2018
2MG-A0204-030		Saudi Arabia	Saudi Arabia	Cluster I (South Asia/Middle East clade)	Cluster I (South Asia/Middle East)	2018

a. All tested isolates were identified by MALDI-TOF MS.
b. Type-strain of *C. auris* (= CBS 10913).

1. Muñoz JF, Gade L, Chow NA, Loparev VN, Juieng P, Berkow EL, et al. 2018. Genomic insights into multidrug-resistance, mating and virulence in Candida auris and related emerging species. Nat Commun 9:5346.

2. Prakash A, Sharma C, Singh A, Kumar Singh P, Kumar A, Hagen F, et al. 2016. Evidence of genotypic diversity among Candida auris isolates by multilocus sequence typing, matrix-assisted laser desorption ionization time-of-flight mass spectrometry and amplified fragment length polymorphism. Clin Microbiol Infect Off Publ Eur Soc Clin Microbiol Infect Dis 22:277.e1–9.

3. de Groot T, Puts Y, Berrio I, Chowdhary A, Meis JF. Development of Candida auris Short Tandem Repeat Typing and Its Application to a Global Collection of Isolates. mBio. 2020;11(1):e02971-19. doi: 10.1128/mBio.02971-19.
